# Homogeneity Enhancement of Mixtures Containing Epoxy Polymer and 100% Reclaimed Asphalt Pavement

**DOI:** 10.3390/polym15214261

**Published:** 2023-10-30

**Authors:** Jun Yang, Xingyu Yi, Huimin Chen, Yiik Diew Wong, Yulou Fan, Wei Huang

**Affiliations:** 1 School of Transportation, Southeast University, Nanjing 210096, China; yixingyu@seu.edu.cn (X.Y.); 220203338@seu.edu.cn (H.C.); yulou_fan@126.com (Y.F.); hhhwei2005@126.com (W.H.); 2 School of Civil and Environmental Engineering, Nanyang Technological University, Singapore 639798, Singapore; cydwong@ntu.edu.sg

**Keywords:** reclaimed asphalt pavement, epoxy resin, asphalt mastic, aggregate, diffusion, homogeneity

## Abstract

The utilization of reclaimed asphalt pavement (RAP) could reduce the cost of pavements containing epoxy polymer (EP) materials. This study was aimed at improving the homogeneity of an EP-reclaimed asphalt mixtures (ERAMs) at both the micro- and meso-scale to provide a reference for an ERAM production process. At the microscale, nanoindentation tests were conducted to characterize the diffusion between the EP and aged asphalt mastic. At the mesoscale, computerized tomography (CT) X-ray scanning and MATLAB analysis were employed to investigate the distribution of the aggregate within the ERAM. The results revealed that mixing temperature played a significant role in the diffusion and distribution between the EP and the aged asphalt mastic, thus impacting the mechanical properties of the material. Heating at 180 °C (the recommended mixing temperature of EP) resulted in a wider blending zone between the EP and the aged asphalt mastic compared to heating at 160 °C (the usual mixing temperature of ordinary reclaimed asphalt mixtures). The overall dispersion of the aggregate in the ERAM exhibited greater homogeneity in the vertical direction than in the horizontal direction. Adjusting the gradation of the RAP was found to be effective in reducing horizontal variability in the distribution of the coarse aggregate, fine aggregate, and air voids in the ERAM. Adjusting the RAP gradation further enhanced the vertical homogeneity in the distribution of the fine aggregate, while its impact on the vertical distribution of the coarse aggregate was minimal. Short-term aging led to increased variability in the distribution of the coarse aggregate, fine aggregate, and air voids within the ERAM. However, adjusting the gradation was effective in mitigating the adverse effects of short-term aging on both horizontal and vertical homogeneity in the aggregate distribution.

## 1. Introduction

Epoxy asphalt (EA) is a modified asphalt consisting primarily of an epoxy polymer (EP) and asphalt [1,2]. EA finds wide application in pavement construction [3], encompassing epoxy asphalt, epoxy mixture concrete, epoxy adhesives, and coatings [4]. Among these, EP belong to one of the most versatile groups of thermosetting polymers, formed by the reaction of epoxy ring functional groups with curing agents [5]. Upon curing, EP forms a dense, cross-linked network structure in a three-dimensional space, with the asphalt phase filling this network structure [6,7,8]. The combination of thermoplastic asphalt with a thermosetting EP not only reduces the brittleness of the EP but also alters the thermoplastic nature of asphalt [9], resulting in the exceptional performance of EA. In comparison to ordinary asphalt mixture materials, epoxy asphalt mixture (EAM) demonstrates enhanced thermal stability, mechanical properties, adhesive properties, corrosion resistance, and chemical resistance [10,11,12,13]. Test results of EAM show significant improvements, with most property metrics being at least 2 to 3 times better and sometimes even more than 10 times better than those of unmodified asphalt mixture [14].

However, the use of epoxy materials in pavement construction can substantially increase initial construction costs [15]. Additionally, the modification of mixing plants to produce EAM incurs additional expenses for the asphalt industry. Nonetheless, due to the outstanding performance of EAM, it is considered a promising material for long-life pavements [8,16]. Several studies have indicated that porous EAM have a life expectancy of approximately 40 years or more [17,18]. Constructing long-life pavements reduces maintenance and rehabilitation costs. Furthermore, in the case of dense-graded EAM, the layer thickness can be reduced by 50–60% while still achieving the same structural functions as those of conventional asphalt mixtures [19]. Consequently, EAM pavements can lower construction costs by reducing the layer thickness. However, there is currently a lack of effective life cycle cost analyses (LCCA) for EAM to demonstrate its economic advantages [20].

The incorporation of reclaimed asphalt pavement (RAP) into EAM presents a promising strategy for reducing the costs of pavement construction and rehabilitation. This approach offers potential cost savings by decreasing the expenses and environmental emissions associated with the extraction, processing, and transportation of raw asphalt and aggregate materials [21,22,23,24]. In the case of ordinary hot-mix asphalt mixture, the use of 100% RAP has the potential to reduce pavement construction costs by 50–70% [25]. The complete utilization of RAP material, with 100% RAP content, in asphalt pavement materials could lead to savings of 18 kg of carbon dioxide emissions per tonne of mixture [25]. Within carbon emission trading systems, reducing carbon emissions translates to cost savings, as enterprises or organizations can generate economic benefits by selling saved carbon emission quotas [26]. Moreover, the use of RAP helps to diminish the volume of material deposited in landfills [27], thereby reducing the disposal costs associated with construction projects. Consequently, the use of reclaimed materials holds significant importance in reducing the overall cost of EAM pavements.

A major concern regarding the utilization of a high proportion of RAP in asphalt mixtures is the insufficient effectiveness of blending and diffusion between the RAP binder mastic and additives, potentially resulting in uneven field performance [28]. Furthermore, the occurrence of particle agglomerations, particularly of smaller-sized RAP particles, has been often observed [28,29,30]. These agglomerations hinder the contact between smaller-sized RAP particles and EP additives, leading to increased aggregate heterogeneity of the mixtures and a reduced degree of blending between the RAP binder mastic and additives [31]. Yi et al. [32] incorporated an EP into 100% RAP and found that the ERAM exhibited noticeably inferior fatigue performance compared to the EAM. They attributed the poorer performance to the low mixing temperature affecting the homogeneity of the mixture. Due to the fact that the preheating temperature of the RAP is lower than the final mixing temperature of the mixture, an ERAM with a higher RAP content may face challenges in reaching the designated mixing temperature within a short mixing duration of the final mixing process. This may cause insufficient mutual diffusion between the epoxy resin and the aged asphalt binder in the RAP. The uncured EP was shown to be unable to adequately penetrate the agglomerates of smaller-sized RAP particles.

The homogeneity of asphalt mixtures containing RAP can be assessed at three different scales: the microscale, the mesoscale, and the macroscale [33]. The microscale homogeneity primarily pertains to the diffusion of the RAP binder mastic and the additives. The mesoscale homogeneity is associated with the distribution of the aggregate. Finally, the macroscale homogeneity is related to the paving and compaction processes. These three scales describe the homogeneity of the asphalt mastic, aggregate, and air voids within the mixture. An optimized mixing procedure for producing an ERAM can enhance the homogeneity at the micro- and meso-scales. Therefore, to improve the performance of the ERAM with 100% RAP and promote the application of RAP in EP-containing pavement materials, this study focuses on enhancing the homogeneity of the material at the asphalt mastic and aggregate levels. At the microscale, the impact of altering the mixing temperature on the diffusion between the EP and the aged asphalt mastic was characterized using a nanoindentation test. At the mesoscale, the effect of adjusting the gradation of the RAP on the distribution of aggregates in the ERAM was investigated using computerized tomography (CT) X-ray scanning. A MATLAB analysis of the internal structure images of the mixture was conducted, along with calculations of homogeneity indices. The findings provide insights into the ERAM production process.

## 2. Materials and Methods

### 2.1. Materials

#### 2.1.1. Asphalt Mastic

The virgin asphalt used in this study was a 60/80 asphalt produced by Jiangsu Alpha (Jiangyin) Asphalt Co. To prepare artificially aged asphalt, standard Rolling Thin Film Oven Test (RTFOT) aging and Pressure Aging Vessel (PAV) aging were conducted according to AASHTO T240 and AASHTO R28, respectively. The instruments are produced by Prentex, Dallas, TX, USA. The basic properties of virgin asphalt and artificially aged asphalt are shown in Table 1. After calculating the content of mineral filler and aged asphalt in the RAP, the artificially aged asphalt was mixed with mineral filler at a ratio of 1:1 to prepare the aged asphalt mastic.

#### 2.1.2. Epoxy Resins and Curing Agent

The bisphenol A epoxy resin and amine curing agent used in this study were produced by a Japanese Taiyu Construction Co., Ltd. (Neyagawa City, Japan). For the uncured EP, the epoxy resin to curing agent ratio was 56:44. After curing in an oven, the tensile strength and elongation of the EP were tested. The technical specifications of the epoxy resin and curing agent are shown in Table 2.

#### 2.1.3. RAP

**Figure 1:**
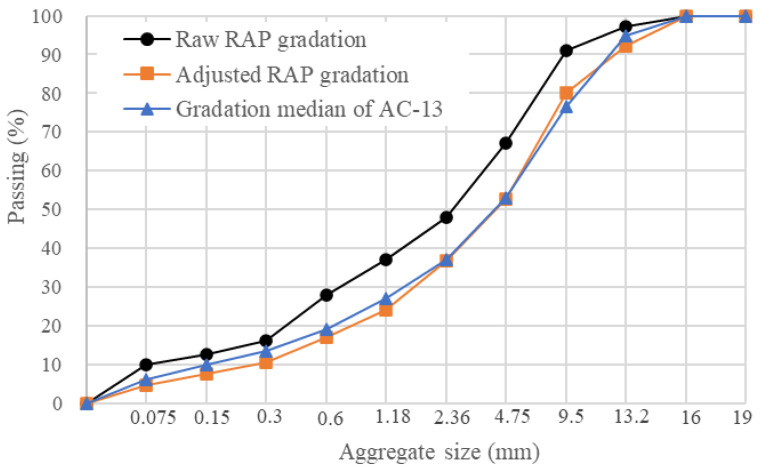
The gradation of RAP and adjusted RAP.

The performance grade of the aged asphalt binder extracted from raw RAP was PG 88-16. The raw RAP contained an average asphalt content of 5%. Notably, the raw RAP consisted of a substantial amount of fine aggregate; its gradation is presented in Figure 1. The gradation of raw RAP is adjusted based on the gradation median of AC-13. To modify the gradation of the RAP, a 4.75 mm sieve was employed to separate raw RAP into two gradations: RAP particles smaller than 4.75 mm (RAP < 4.75 mm) and RAP particles larger than 4.75 mm (RAP > 4.75 mm). After calculation, an adjusted RAP was created by combining 25% of the RAP < 4.75 mm and 75% of the RAP > 4.75 mm. The gradation of adjusted RAP is also presented in Figure 1. The gradation of adjusted RAP is very close to the gradation median of AC-13.

### 2.2. Methods

#### 2.2.1. Preparation of Nano-Indentation Test Samples

**Figure 2:**
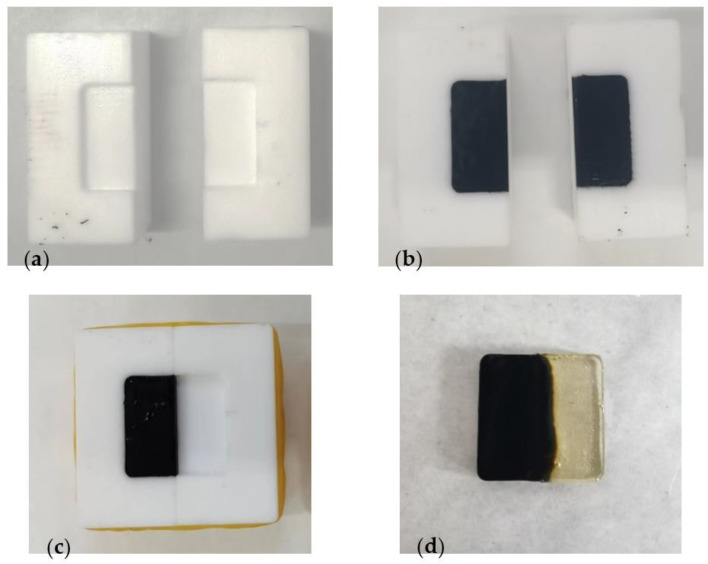
Steps for making nanoindentation test samples: (**a**) clean the mould; (**b**) pour the aged asphalt mastic in the mould; (**c**) fix the mould; (**d**) pour the EP and finish the curing.

In this study, a newly developed test was used to simulate the mixing of EP and RAP asphalt mastic. A custom-made mold was utilized to facilitate the diffusion of EP and asphalt mastic. The mold is depicted in Figure 2a. Melted artificially aged asphalt mastic was poured into one side of the mold, as shown in Figure 2b,c. After allowing it to cool, the uncured EP was poured into the other side, ensuring that the heights on both sides were level. The diffusion of the two components was achieved by heating the mold in an oven at the experimental temperature for 10 min to simulate the mixing temperature of ERAM. Finally, the samples were cured by placing them in an oven at 60 °C for four days. The cured sample is illustrated in Figure 2d. The dimensions of the cured sample were 20 mm along the side length and 2 mm in thickness.

In order to prevent the asphalt in the RAP from re-aging during the recycling mixing process, a final mixing temperature of 160 °C was used, as in previous research [32]. Since RAP was only preheated at 120 °C, when 100% RAP was added to the mixing machine, the actual final mixing temperature was less than 160 °C. This was because the mixing time for RAP and EP was only 90 s. Although the mixing machine was set to a target final mixing temperature of 160 °C, the actual mix temperature may have been slightly lower due to the fact that the RAP temperature was only 120 °C. This may have limited the modification effect of the epoxy polymer to a certain extent. Therefore, increasing the mixing temperature may positively affect the performance of the epoxy reclaimed mixture. The epoxy polymer (EP) used in this study was a high-temperature mixing epoxy resin material. In the case of the virgin epoxy asphalt mixture without any RAP, the typical mixing temperature ranges from 170 °C to 190 °C [34]. Therefore, the selected temperature for the diffusion process was 180 °C. Meanwhile, 160 °C was also used as a control group.

#### 2.2.2. Nanoindentation Test

Nanoindentation is an innovative technique for testing the micro-mechanical properties of materials. It involves conducting indentation tests with a nanoscale indenter and extracting the sample’s micro-mechanical properties using relevant mechanical models. This technique enables accurate measurements to be made of the fundamental mechanical properties of different phases within heterogeneous mixtures using small sample volumes [35]. At the microscale, the indenter probes the smooth surface of the sample, and the load and depth during indentation are recorded, resulting in a load–depth curve. Subsequently, various computational models are employed to analyze the experimental curve and extract parameters such as hardness and modulus. The test consists of three stages: the loading stage, the holding stage, and the unloading stage. The holding stage refers to the phase where the load remains constant while the indentation depth increases.

Nanoindentation testing was conducted to assess the mechanical properties of the diffusion between the EP and the aged asphalt mastic. The study employed a System 1 nanoindenter produced by Micro Materials, USA. A Berkovich indenter was used with the parameters specified in Table 3. The maximum load was set to 0.1 mN, with a loading/unloading rate of 0.01 mN/s and a holding time of 200 s.

**Figure 3:**
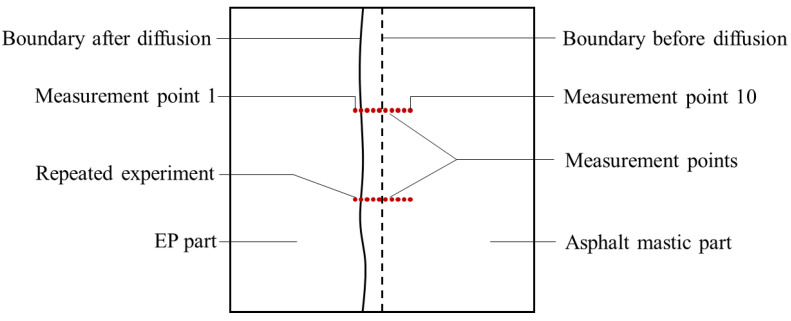
Schematic diagram of the measurement points.

For each asphalt mastic sample, 20 measurement points were set along two lines perpendicular to the interface between the EP and asphalt mastic, as shown in Figure 3. Ten measurement points were evenly distributed along each line, with a spacing of 500 μm. The measurement points were numbered from 1 to 10 in a line. The measurement points on the other line were repeated. The data measured at the position with the same point number on each line were averaged. Due to the distinct color difference between the EP and asphalt mastic parts, the boundary between the amber and black regions of the sample could be identified under a microscope. A displacement of approximately 500 μm from the boundary toward the EP part was used to determine the starting position of measurement point 1, ensuring that it was located within the EP phase. Nanoindentation tests were performed on EP-mastic samples prepared at two temperatures. The microscale modulus was extracted from the indentation tests, and the extent of diffusion between the asphalt EP and aged asphalt mastic was evaluated by examining the variation in the elastic modulus along the line of measurement points.

#### 2.2.3. Preparation of ERAM Mixture Samples

**Figure 4:**
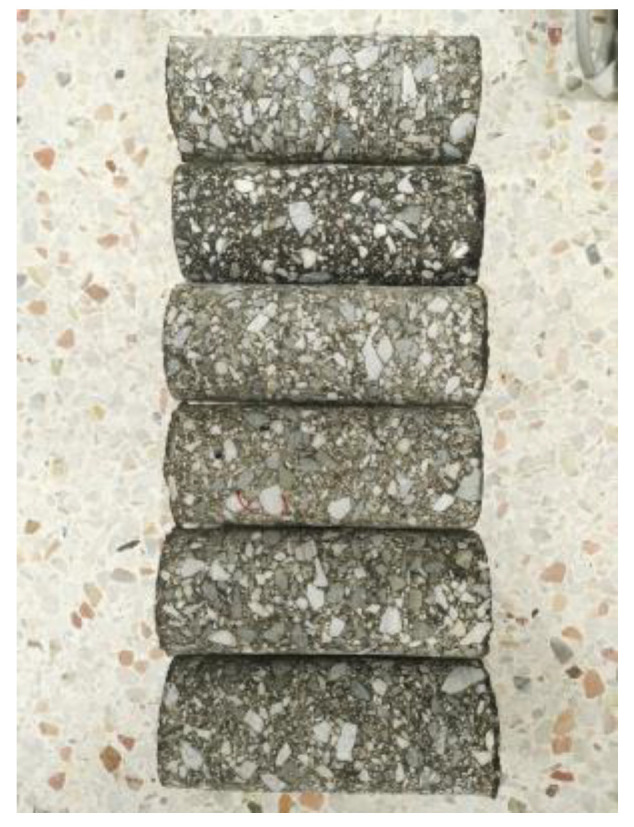
70 mm-diameter, 160 mm-thick cored specimens.

In this study, ERAM was prepared using both raw RAP and adjusted RAP. Initially, the RAPs were dried in an oven at 120 °C for 2 h. Subsequently, uncured EP was added to the mixing machine with a mass ratio of 3:7 between EP and aged asphalt binder, based on a previous study [36]. The RAPs were then introduced into the machine and mixed. The resulting mixture was placed into the mold (of 150 diameter and 160 depth) and compacted using a gyratory compactor. Based on previous experience [32], the compaction process was repeated until the void content has been determined to be 2%. After compaction, the specimens were transferred to an oven and heated at 60 °C for 4 days to facilitate curing. Finally, cylindrical specimens, each with a diameter of 70 mm and a height of 160 mm, were obtained by coring the compacted specimens along the central portions, as shown in Figure 4.

Asphalt in the mixture used in pavement construction undergoes short-term aging during the mixing, transportation, and paving processes. This aging leads to an increase in the viscosity of the asphalt, which hinders the relative sliding between particles in the mixture and consequently affects its homogeneity. To simulate the effect of short-term aging on the mixture, a portion of the uncompacted mixture was subjected to ventilation and heating for 4 h at 135 °C in an oven. The labels of the mixture samples are shown in Table 4, wherein three replicate specimens were prepared for each asphalt mixture.

#### 2.2.4. X-ray Computerized Tomography (CT) Test and Analysis

CT scanning technology is a non-destructive technique for the acquisition of internal structural information about asphalt mixtures. To this end, it exploits the different X-ray absorption capabilities of various constituent materials within the mixture. This technique is suitable for analyzing the distribution and homogeneity of coarse and fine aggregate, as well as air voids within the asphalt mixture along both the horizontal and vertical directions [37]. Since the grayscale values in the original CT images are related to the material density, it is relatively easy to differentiate between the void phase (appearing as black), the aggregate phase (appearing as lighter shades), and the asphalt mastic phase (appearing as darker shades). MATLAB programs were adopted to process the original CT images, thereby extracting spatial distribution information for each constituent material and quantitatively calculating spatial indices to evaluate the homogeneity of the mixture.

**Figure 5:**
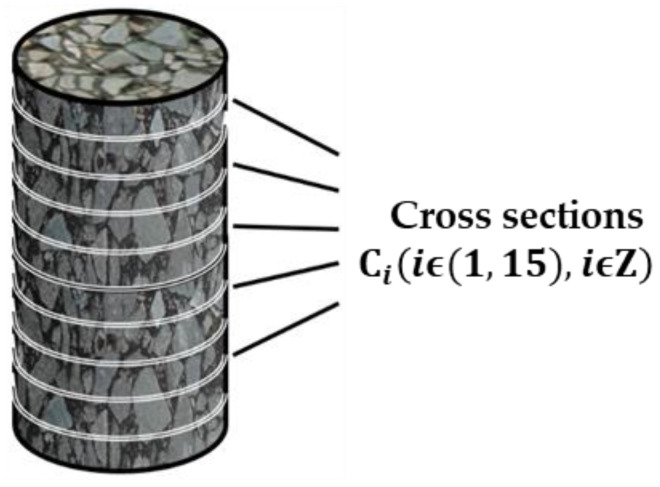
Schematic diagram of cross sections.

**Figure 6:**
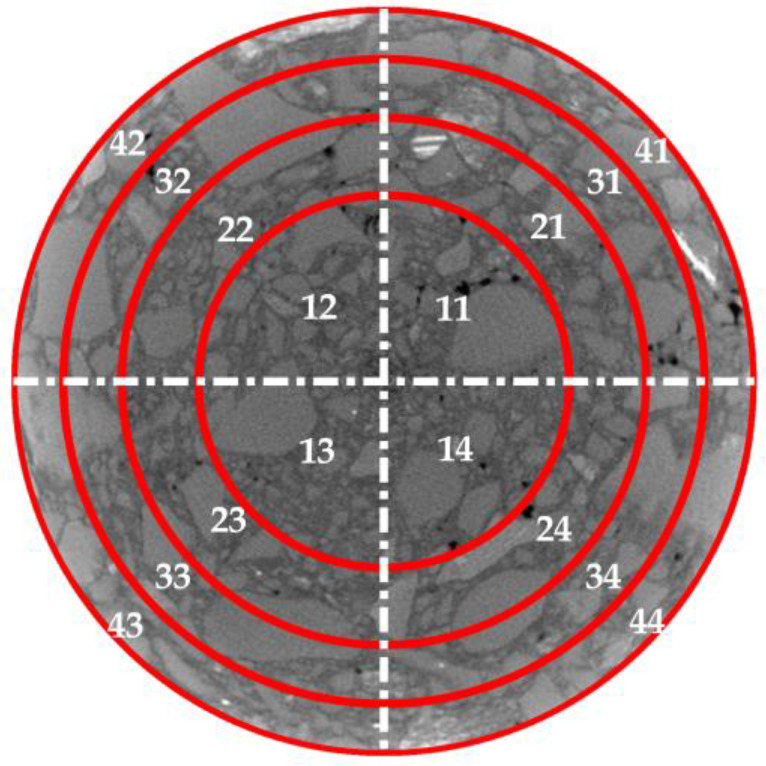
Division of section.

**Figure 7:**
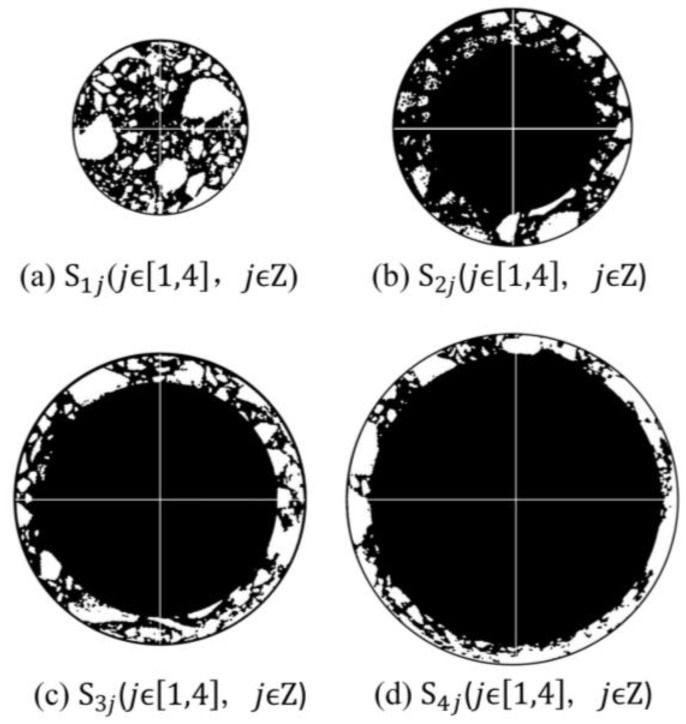
MATLAB image processing results of different partitioned regions.

A longitudinal cross-section was taken along the cylindrical specimens at intervals of 1 cm, resulting in a total of 15 cross-sections, labeled as $C_{i}\left(i\epsilon \left[1,15\right],i\epsilon Z\right)$, as shown in Figure 5. In this study, a German YXLON X-ray CT scanner was employed to perform CT scans on different cross-sections of the ERAM specimens. The obtained CT images were divided into equal-area annular and circular regions. Each annular region was further divided into four equal quadrants based on the coordinate axes, resulting in distinct regions named $S_{jj}(j\epsilon \left[1,4\right],j\epsilon Z)$, as illustrated in Figure 6. The areas of each region were identical. MATLAB was used to process the internal structure images of the asphalt mixture, as shown in Figure 7. Subsequently, the areas of coarse aggregate (particle size > 4.75 mm), fine aggregate (particle size < 4.75 mm), and air voids were determined within each region Sjj, and the homogeneity indices of each constituent were calculated. The study compared the homogeneity of the mixture from both the horizontal and vertical perspectives.

To assess horizontal homogeneity, the areas of coarse aggregate, fine aggregate, and voids within each region $S_{jj}(j\epsilon (1,4),j\epsilon Z)$ in a single cross-section were calculated. Subsequently, the coefficient of variation, $D_{H}$, was determined for each constituent in different regions $S_{jj}(j\epsilon \left[1,4\right],j\epsilon Z)$. The mean coefficient of variation, ${\bar{D}}_{H}$, for the 15 cross-sections was computed by averaging the individual coefficients of variation, $D_{Hk}\left(k\epsilon \left[1,15\right],k\epsilon Z\right)$, using Equation (1):

(1) $${\bar{D}}_{H}=\frac{\sum D_{Hk}}{15}\left(k\epsilon (1,15),k\epsilon Z\right)$$

where ${\bar{D}}_{H}$ serves as an indicator of the horizontal (cross-sectional) homogeneity.

For instance, in the case of cross-section $C_{1}$, the coefficients of variation for areas of coarse aggregate, fine aggregate, and air voids, denoted as $D_{H1}$, were first calculated as presented in Table 5. Subsequently, coefficients of variation, $D_{Hk}\left(k\epsilon (1,15),k\epsilon Z\right)$, and their mean value, ${\bar{D}}_{H}$, were computed across all the cross-sections $C_{i}\left(i\epsilon (1,15),i\epsilon Z\right)$ of the specimen, as shown in Table 6.

For vertical homogeneity, the areas of coarse aggregate, fine aggregate, and air void phases were computed for each cross-section. The coefficient of variation, $D_{V}$, calculated from the variations across the 15 cross-sections, served as an indicator to assess the degree of vertical distribution segregation. Additionally, the variations of $D_{Hk}\left(k\epsilon \left[1,15\right],k\epsilon Z\right)$ along the vertical direction were analyzed to examine the vertical distribution of each phase in ERAM.

## 3. Results and Discussion

### 3.1. Homogeneity of Asphalt Mastic

**Figure 8:**
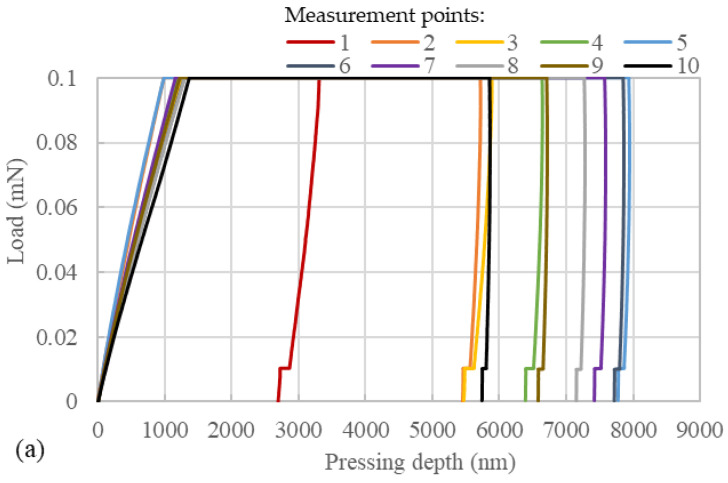

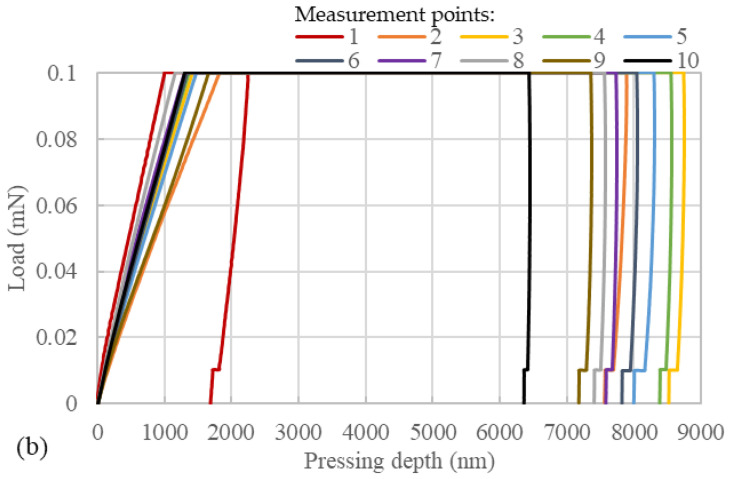
Load–depth curve of the samples with the diffusion temperature of (**a**) 160 °C and (**b**) 180 °C.

Nanoindentation tests were performed on samples prepared at two diffusion temperatures; the load–depth curves are shown in Figure 8. It can be observed that for both samples, the maximum indentation depth was smallest at measurement point 1. This indicates that pure EP exhibited a high resistance to deformation. As the measurement points gradually approached the asphalt mastic part from the EP part, the resistance to deformation of the samples showed a decreasing trend, followed by an increasing trend. In Figure 8a, measurement point 5 exhibited the greatest maximum indentation depth, while in Figure 8b, measurement point 3 showed the highest maximum indentation depth. These findings suggest that the mechanical properties of EP and aged asphalt mastic underwent changes upon their mutual diffusion. Prior to measurement point 5 for the sample with a diffusion temperature of 160 °C and measurement point 3 for the sample with a diffusion temperature of 180 °C, both samples exhibited a decrease in resistance to deformation. After these measurement points, the maximum indentation depth gradually increased as the measurement point number increased up to measurement point 10. The indentation depth at measurement point 10 was significantly higher than that of measurement point 1. This indicates that an increase in aged asphalt mastic concentration led to a reduction in the resistance to deformation of the samples to some extent.

Notably, measurement points 1 and 10 in the sample with 180 °C diffusion temperature exhibited smaller maximum indentation depths compared to the remaining measurement points. Similarly, in the sample with a diffusion temperature of 160 °C, measurement points 1 and 10 exhibited smaller maximum indentation depths than measurement points 4–9. In other words, the mechanical properties of the blending zone did not fall between those of EP and asphalt. This suggests that the diffusion between EP and asphalt mastic was not a simple blending, and the irregular network structure formed by partially diffused EP significantly affected the mechanical properties of the samples. In addition, the maximum indentation depth differed between the two samples, with the 160-°C sample having a maximum indentation depth of less than 8000 nm, while the 180-°C sample approached 9000 nm. This indicates that the diffusion temperature between EP and asphalt mastic affected the distribution of EP, thereby significantly influencing the material’s mechanical properties.

**Figure 9:**
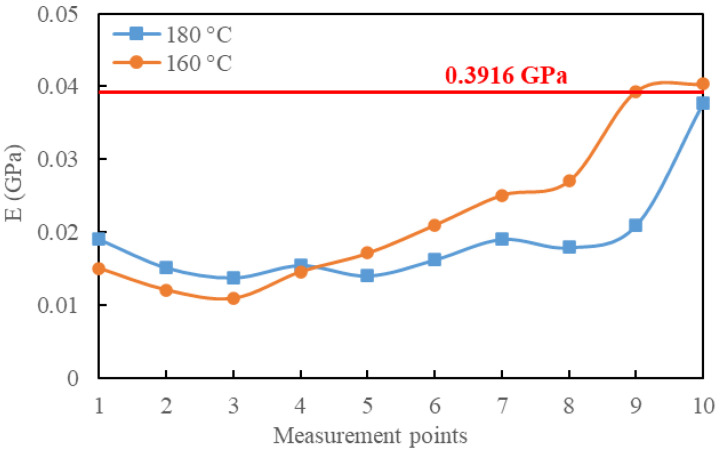
Elasticity module E of samples at two diffusion temperatures.

Based on the load–depth curves obtained from different measurement points, the elastic modulus E of the two samples was calculated, as shown in Figure 9. It can be observed that there were significant modulus variations at different measurement points, indicating a micro-mechanical property transition in the diffusion region between the EP and asphalt mastic. Both samples exhibited a decreasing trend in E for the initial measurement points, followed by an increase. Several nanoindentation tests were conducted on pure asphalt mastic samples, and the E of pure asphalt mastic samples was calculated based on load–unload curves. The average E of the pure asphalt mastic was measured to be 0.03916 GPa with a coefficient of variation of 6.8%. By comparing this average modulus with the two curves in Figure 9, it can be observed that the 160-°C sample reached this modulus level at measurement point 9, while the 180-°C sample approached this level at measurement point 10. The measurement point 1 for both samples was at the same distance from the EP-mastic boundary. This indicates that increasing the fusion temperature to 180 °C results in a wider diffusion region. At 180 °C, the diffusion between the EP and asphalt mastic was more thorough. This means that mixing RAP and EP at 180 °C will result in a better homogeneity of the binder mastic in ERAM. By enhancing diffusion, the viscosity of asphalt in the agglomerations may be reduced, thereby increasing the possibility of dispersion in the agglomerations.

### 3.2. Homogeneity of Aggregate

#### 3.2.1. Horizontal Homogeneity of Aggregate

**Figure 10:**
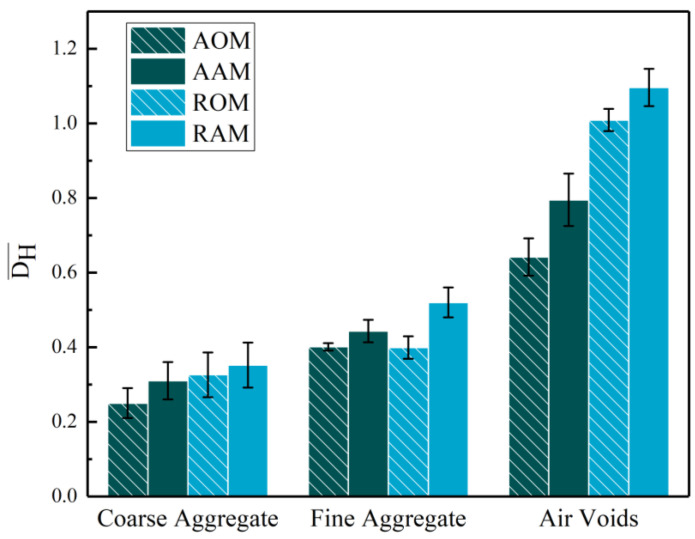
Mean coefficient of variation ${\bar{D}}_{H}$ of coarse aggregate, fine aggregate, and air voids.

The CT images of the four types of mixture specimens were processed, and the mean value ${\bar{D}}_{H}$ of the $D_{H}$ for all 15 cross-sections of the parallel specimens was calculated, as shown in Figure 10. It can be observed that the mean coefficient of variation ${\bar{D}}_{H}$ for coarse aggregate was smaller than that of fine aggregate. This indicates that the skeletal structure of coarse aggregate was distributed in a more homogeneous manner, while the fine aggregate exhibited poorer homogeneity due to agglomeration. The ${\bar{D}}_{H}$ for air voids was the highest, indicating the highly uneven dispersion within the cross-sections. This could be attributed to the relatively low overall void content of the specimens and the small area of the divided regions, which resulted in fewer voids in certain divided areas, thereby increasing the variability in the size of air voids between different regions. The average variation coefficients, ${\bar{D}}_{H}$, for coarse aggregate, fine aggregate, and air voids in the adjusted RAP were all smaller than those in the raw RAP. This indicates that the use of adjusted RAP effectively reduced the horizontal distribution variability of coarse aggregate, fine aggregate, and air voids in ERAM.

Short-term aging increased the horizontal distribution variability of coarse aggregate, fine aggregate, and air voids in ERAM, with the most significant impact observed on the distribution of air voids. This indicates that during the short-term aging process, a certain degree of EP curing occurred, while the aged asphalt in the RAP underwent a certain level of re-aging, leading to a noticeable increase in the viscosity of the binder in ERAM. This reduced the inter-particle sliding in the RAP and affected the compaction of ERAM, resulting in the increased disparity of air voids.

#### 3.2.2. Vertical Homogeneity of Aggregate

**Figure 11:**
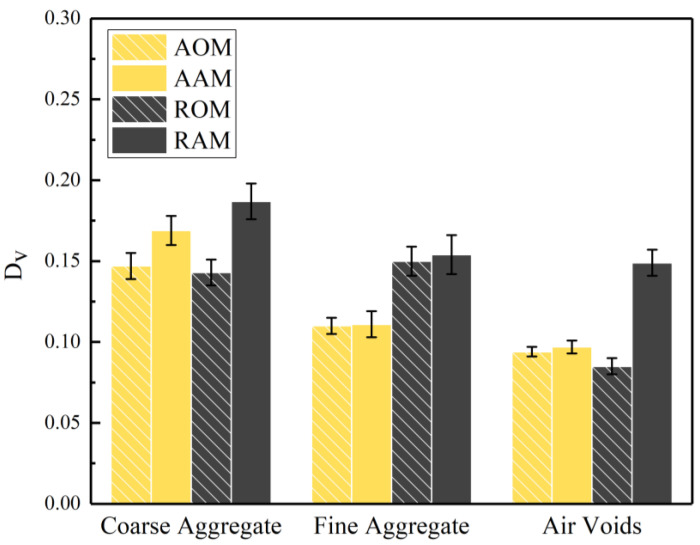
The coefficient of variation $D_{V}$ of coarse aggregate, fine aggregate, and air voids.

The areas of coarse aggregate, fine aggregate, and air voids were calculated from the CT images of the 15 cross-sections, and the vertical coefficient of variation $D_{V}$ for each phase was determined, as shown in Figure 11. It can be observed that compared to ${\bar{D}}_{H}$, the $D_{V}$ was relatively small, indicating a relatively homogeneous overall dispersion of ERAM in the vertical direction. The $D_{V}$ for the coarse aggregate was larger than that for the fine aggregate phase. This may be attributed to the discontinuity of the coarse aggregate skeletal structure in the mixture, resulting from its segmentation by the fine aggregate.

Adjusting the gradation of RAP can effectively reduce the $D_{V}$ of the fine aggregate in the mixture while having a minimal impact on the $D_{V}$ of the coarse aggregate. This is because, in adjusted RAP, there was a higher proportion of coarse aggregate and a lower proportion of fine aggregate, thereby reducing the phenomenon of fine aggregate accumulation at the bottom of the specimen. Additionally, the abundant coarse aggregate skeletal structure restricted the downward movement of some fine aggregate, resulting in less pronounced vertical segregation between coarse and fine aggregates.

After short-term aging, there was a certain degree of increase in $D_{V}$ for the coarse aggregate phase, fine aggregate phase, and air void phase. The influence of short-term aging on the $D_{V}$ of the fine aggregate in all ERAM mixtures was not significant, suggesting that the agglomeration of the fine aggregate may have already occurred during the mixing process. For the AAM and AOM, the $D_{V}$ for each phase was smaller compared to the RAM and ROM, respectively. Furthermore, the differences in $D_{V}$ between AAM and AOM were smaller compared to RAM and ROM. This indicates that adjusting the gradation can effectively improve the vertical homogeneity of ERAM and reduce the adverse effects of short-term aging on its homogeneity.

**Figure 12:**
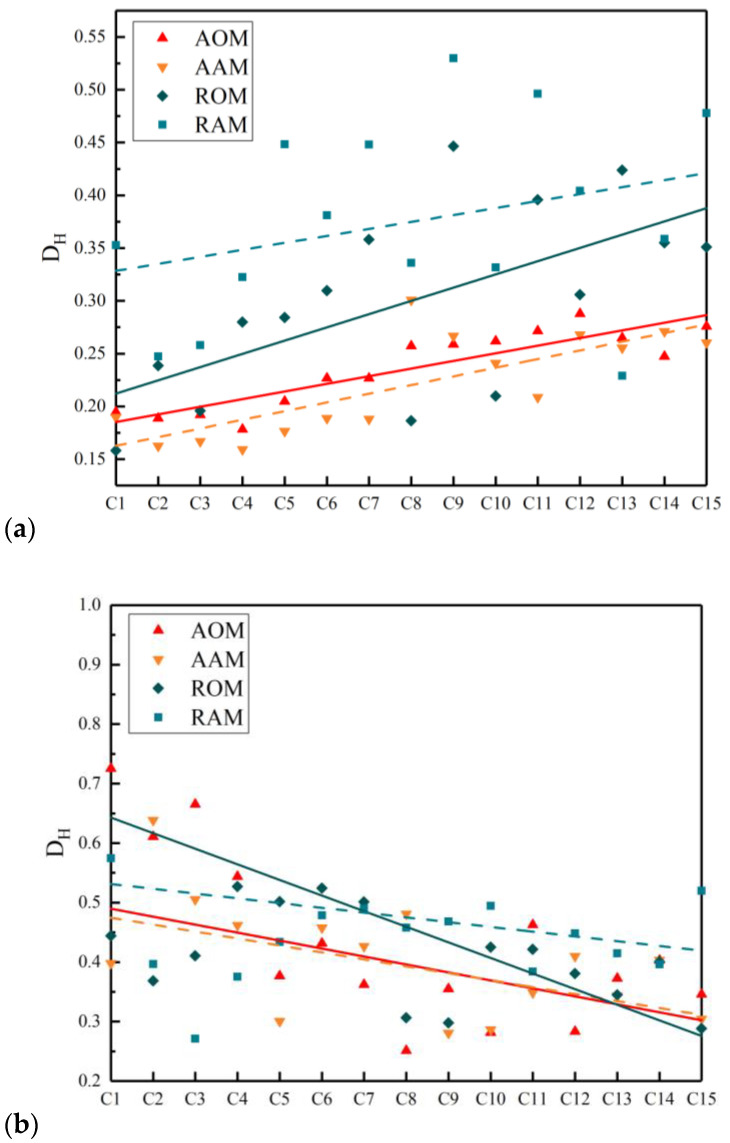

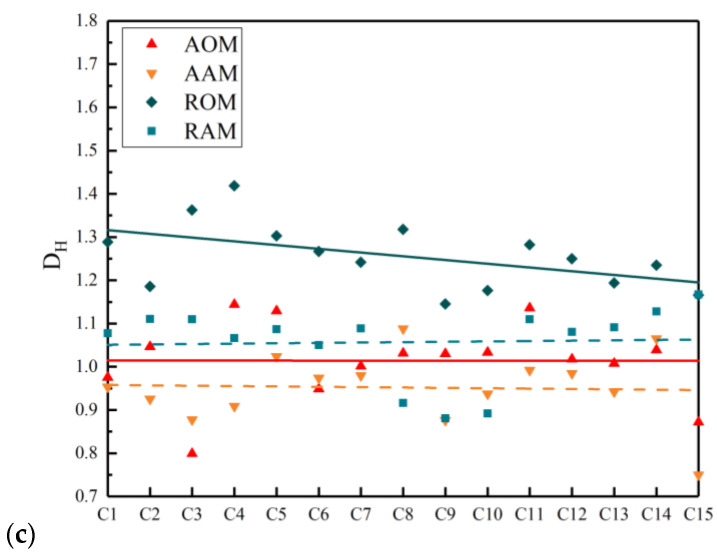
The coefficient of variation $D_{H}$ of (**a**) coarse aggregate, (**b**) fine aggregate, and (**c**) air voids.

The $D_{H}$ values for each cross-section are exhibited in Figure 12, where a trend line is added. Realisations represent original mixtures and dashed lines represent short-term aged mixtures. It can be observed that for the fine aggregate, coarse aggregate, and air voids, the vertical variations of $D_{H}$ in each cross-section of AAM and AOM were smaller compared to RAM and ROM. This indicates that adjusting the gradation was beneficial to optimizing the vertical distribution homogeneity of fine aggregate, coarse aggregate, and air voids in ERAM. Additionally, the distance between the $D_{H}$ trend lines of the three phases in AAM and AOM was significantly lower than that in RAM and ROM. This indicates that adjusting the RAP gradation was also advantageous in reducing the impact of short-term aging on the vertical distribution homogeneity of ERAM.

It is noteworthy that the $D_{H}$ curves for coarse aggregate exhibited an increasing trend, while the $D_{H}$ curves for fine aggregate showed a decreasing trend among the four mixtures. This suggests that the vertical distribution homogeneity of coarse aggregate was poorer at the bottom of the specimen, while the vertical distribution homogeneity of fine aggregate was poorer at the top. This implies that fine aggregate accumulated at the bottom of the specimen, leading to a certain degree of segregation between coarse and fine aggregates.

## 4. Conclusions

To promote the application of RAP in ERAM and provide a reference for the production process of ERAM, this study focused on improving the homogeneity at both the asphalt mastic and aggregate levels. At the microscale, the diffusion between EP and aged asphalt mastic was characterized using nanoindentation tests. At the mesoscale, computerized tomography (CT) X-ray scanning and MATLAB analysis were employed to investigate the aggregate distribution in ERAM. The following conclusions can be drawn from the data analyses:

The mixing temperature influenced the diffusion between EP and asphalt mastic as well as the distribution of EP, significantly impacting the mechanical properties of the material. Compared to heating at 160 °C, a wider blending zone between the EP and asphalt mastic appeared when heating at 180 °C.

The use of adjusted RAP effectively reduced the horizontal distribution variability of coarse aggregate, fine aggregate, and air voids in ERAM. The overall dispersion of the aggregate in the ERAM was more homogeneous in the vertical direction than in the horizontal direction. Adjusting the RAP gradation improved the vertical distribution homogeneity of the fine aggregate in ERAM, while it had little influence on the vertical distribution homogeneity of the coarse aggregate.

Short-term aging increased the distribution variability of the coarse aggregate, fine aggregate phase, and air voids in ERAM, with the most pronounced impact being observed on the distribution of air voids. Adjusting the gradation was found to be effective at mitigating the adverse effects of short-term aging on vertical distribution homogeneity.

## Figures and Tables

**Table 1:** Basic properties of virgin asphalt and aged asphalt.

Materials	Properties				
	Penetration (0.1 mm)	Softening Point (°C)	Ductility (cm, 15 °C)	Viscosity (Pa·s, 135 °C)	Performance Grade
Virgin asphalt	71	47.5	160.1	0.6	PG 64–22
Aged asphalt	23	66.5	5.2	1.83	PG 88–16

**Table 2:** Basic properties of epoxy resin and curing agent.

Items	Materials	Values
Density (g/cm^3^)	Epoxy resin (before curing)	1.118
	Curing agent (before curing)	0.852
Viscosity(cps, 25 °C)	Epoxy resin (before curing)	2.118
	Curing agent (before curing)	0.242
Tensile strength (MPa)	Epoxy resin components after curing	1.65
Elongation (%)		191

**Table 3:** Berkovich indenter specifications.

Probe Type	Half-Open Angle (°)	Effective Cone Angle (°)	Geometric Correction Factor	Young’s Modulus (GPa)	Poisson Ratio
Berkovich	65.27	70.3	1.034	1141	0.07

**Table 4:** Labels of mixture samples.

Gradation	Aging Degree	ERAM Labels
Raw RAP	Original	ROM
	Shot-term aging	RAM
Adjusted RAP	Original	AOM
	Shot-term aging	AAM

**Table 5:** Calculation process for $D_{H1}$.

Regions	$\mathrm{Area}\;({mm}^{2}$)		
	Coarse Aggregate Phase	Fine Aggregate Phase	Air Void Phase
$S_{11}$	712.32	21.82	5.36
$S_{12}$	672.45	8.84	4.28
$S_{13}$	730.82	4.57	3.91
$S_{14}$	744.58	8.13	1.96
$S_{21}$	463.55	48.16	12.02
$S_{22}$	415.39	69.05	6.50
$S_{23}$	347.70	102.32	8.05
$S_{24}$	382.89	110.11	3.13
$S_{31}$	543.78	71.38	7.64
$S_{32}$	626.09	30.76	1.94
$S_{33}$	366.32	77.63	22.70
$S_{34}$	579.56	41.55	2.97
$S_{41}$	560.15	64.47	4.39
$S_{42}$	460.97	49.62	2.09
$S_{43}$	341.28	36.68	23.94
$S_{44}$	483.88	102.07	2.05
Standard deviation	135.62	33.21	6.70
Average value	526.98	52.95	7.06
Coefficient of $\mathrm{variation}\;D_{H1}$	0.26	0.63	0.95

**Table 6:** Calculation process for ${\bar{D}}_{H}$.

Coefficient of Variation	Coarse Aggregate Phase	Fine Aggregate Phase	Air Void Phase
$D_{H1}$	0.26	0.63	0.95
$D_{H2}$	0.24	0.44	1.06
$D_{H3}$	0.3	0.61	1.10
$D_{H4}$	0.34	0.63	1.06
$D_{H5}$	0.36	0.67	1.06
$D_{H6}$	0.39	0.59	1.01
$D_{H7}$	0.33	0.55	1.04
$D_{H8}$	0.37	0.54	1.06
$D_{H9}$	0.4	0.62	0.96
$D_{H10}$	0.41	0.55	0.99
$D_{H11}$	0.38	0.45	1.00
$D_{H12}$	0.40	0.65	0.94
$D_{H13}$	0.38	0.49	1.04
$D_{H14}$	0.41	0.53	1.04
$D_{H15}$	0.39	0.62	0.97
$\mathrm{Mean}\;\mathrm{value}\;{\bar{D}}_{H}$	0.36	0.57	1.01

## Data Availability

The data presented in this study are available upon request from the corresponding author.
